# Dissociation of early and late face-related processes in autism spectrum disorder and Williams syndrome

**DOI:** 10.1186/s13023-022-02395-6

**Published:** 2022-06-22

**Authors:** Alice Gomez, Guillaume Lio, Manuela Costa, Angela Sirigu, Caroline Demily

**Affiliations:** 1 Institut Des Sciences, Cognitives Marc Jeannerod, Centre National de La Recherche Scientifique, 67 boulevard Pinel, 69500 Bron, France; 2 Claude Bernard University Lyon, Lyon, France; 3 Reference Center for Rare Diseases With Psychiatric Phenotype Génopsy, Le Vinatier Hospital, Bron, France; 4 iMIND Excellence Center for Autism and Neurodevelopmental Disorders, Lyon, France; 5 Present Address: Laboratory for Clinical Neuroscience, Center for Biomedical Technology, University Politécnica de Madrid, Madrid, Spain; 6 Present Address: Lyon Neuroscience Research Center (CRNL), Inserm U1028, CNRS UMR5292, UCBL1, UJM, Lyon, France

**Keywords:** Eye sensitive, Facial features, Fusiform face area, Social brain, Superior Temporal Sulcus

## Abstract

**Background:**

Williams syndrome (WS) and Autism Spectrum Disorders (ASD) are neurodevelopmental conditions associated with atypical but opposite face-to-face interactions patterns: WS patients overly stare at others, ASD individuals escape eye contact. Whether these behaviors result from dissociable visual processes within the occipito-temporal pathways is unknown.

Using high-density electroencephalography, multivariate signal processing algorithms and a protocol designed to identify and extract evoked activities sensitive to facial cues, we investigated how WS (N = 14), ASD (N = 14) and neurotypical subjects (N = 14) decode the information content of a face stimulus.

**Results:**

We found two neural components in neurotypical participants, both strongest when the eye region was projected onto the subject's fovea, simulating a direct eye contact situation, and weakest over more distant regions, reaching a minimum when the focused region was outside the stimulus face. The first component peaks at 170 ms, an early signal known to be implicated in low-level face features. The second is identified later, 260 ms post-stimulus onset and is implicated in decoding salient face social cues.

Remarkably, both components were found distinctly impaired and preserved in WS and ASD. In WS, we could weakly decode the 170 ms signal based on our regressor relative to facial features, probably due to their relatively poor ability to process faces’ morphology, while the late 260 ms component was highly significant. The reverse pattern was observed in ASD participants who showed neurotypical like early 170 ms evoked activity but impaired late evoked 260 ms signal.

**Conclusions:**

Our study reveals a dissociation between WS and ASD patients and points at different neural origins for their social impairments.

## Introduction

WS is a rare neurodevelopmental disorder, with a prevalence of 1 in 7500, caused by a hemizygous deletion of approximately 25 genes on the 7q11.23 chromosomal region Korenberg et al, [1] Stromme, Bjørnstad & Ramstad, [2] resulting in a phenotype comprised of medical, cognitive, affective, and neurophysiological impairments Bellugi et al, [3]. A core behavioral component of this syndrome is increased motivation for social interaction with an apparent lack of fear of strangers [4, 5]. Patients with WS have been described as acting as if “everybody in the world is their friend” [4] and their appetitive drive for social interaction and social closeness with other people is commonly likened to hypersociability [6].

ASD is a neuro-developmental disorder characterized by deficits in social communication and social reciprocity, as well as repetitive and stereotyped behaviors, with an approximate prevalence of 1 in 100 [7]. Despite extensive research into the biological factors underlying its pathology, behavioral observations remain the principal method of diagnosis [8]. Social deficits, most notably a failure to attend preferentially to the eyes of others, are signs of autism that are observable as early as the first months of life [9, 10].

Whereas patients with ASD struggle with social interaction and making eye contact, patients with WS seek to do both. Specifically, during social interaction, patients with WS appear to exhibit “face fascination” Jarvinen et al, [11] from infancy [12]. Relative to more general forms of visuo-spatial processing, WS patients demonstrate comparative strengths in face processing Bellugi et al, [13] (Paul et al., [14]). However, studies have also noted atypical processing in WS patients of eye and mouth regions of the face. Overall, face scanning patterns of individuals with WS differ from those produced by ASD patients. Patients with WS show an increased preference for eyes over the mouth region [15, 16] in upright faces [17], while patients with ASD fail to attend to the eye region of other faces [9, 10]. This pattern of dissociation in face processing between patients with ASD and patients with WS is intriguing, especially considering that face-to-face interaction is a critical ability on which future social interest and social skills are based (e.g., [18–20].

Faces are multidimensional visual stimuli offering a rich variety of information to observers [21]. The multidimensional nature of this information is key to social interaction. Faces not only convey permanent and stable information such as gender (masculine or feminine), race (e.g., Chinese or Caucasian), identity (e.g., John or Mary), but also dynamic and transient information such as emotional expression, direction of attention, and intention [22].

The neurobiological substrate of these complex cognitive processes relies on at least three brain structures: the occipital (OFA) and fusiform face areas (FFA) and the posterior superior temporal sulcus (STS, [23].) Using electroencephalography, the N170, a negative potential that peaks at 170 ms has been shown to be elicited by faces [24] and by face components, especially the eyes Bentin [25].

Along the occipito-temporal visual stream the STS plays a key role in the human face-perception system, but it is also one of the key components of the ‘social brain’. Indeed, brain regions in and around the superior temporal sulcus of both hemispheres may be involved in the analysis of actual or implied facial movements and related cues that provide socially relevant information, such as emotional expression [26–30] and gaze direction [31].

The superior temporal sulcus has also been hypothesized as a key structure associated with the social-interaction deficits that are typical in patients with ASD [8, 32, 33]. Moreover, although WS patients show an overall reduction in brain volume [34–36], cortical thickness of the superior temporal gyrus has been found increased compared to the neurotypical population [37]. Specifically, this hypothesis points to the STS as playing a role in the early stages of visual processing analysis of social cues in ASD and WS.

In the present study, using the face perception task developed by Lio et al. [38], we asked whether the atypical neural signal observed in patients with ASD in the STS might also be observed in WS. Overall, we sought to determine whether the opposite nature of face-related behaviors observed in both conditions can also be reflected at the neural level.

In the present study, we examined the high-density EEG (Electro-Encephalography) brain activity of WS patients compared with data from a group of neurotypical subjects and a group of patients with ASD previously reported by Lio et al. [38] during a face gender discrimination task.

(A) **Facial-cue task** Participants were instructed to focus on a fixation cross and were then presented with a face stimulus masked by a Gaussian apodization window centered around the fixation cross (FWHM = 10°). The focused face area at the center of the screen was randomly drawn from a uniform distribution among 25 predetermined locations. A question mark was presented at random points (every 7 ± 3 trials, at which point, participants were required to determine the gender of the last face stimulus that was viewed (left/right button press). (**B**) **Analysis 1.** The first analysis used a strong spatio-temporal a priori to build a map of cortical reactivity according to the face area that was focused on by each participant. Cortical reactivity was evoked in the region of the STS occurring 200-300 ms after stimulus onset, as in Lio et al. The purpose of this analysis was to determine whether the neurotypical response to eye contact in this source was preserved in WS. (**C**) **Analysis 2.** The second analysis aimed to identify changes in cortical reactivity associated with a “facial cue” regressor over time (built from the Lio et al. Exp 0.2 study, see method), and to identify when in time EEG activity can decode the “facial cue” map. This spatial regressor predicts a maximum evoked activity when participants focus on facial cues, with a progressive decay in response to other parts of the face and a minimum activity outside the face, as in the neurotypical population (See Fig. 3A for details).

Two sets of EEG analysis were performed. First, in a spatio-temporal a priori analysis we built a spatial filter from the STS source identified at 240 ms by Lio et al. [38] and used it to extract evoked single-trial activity. Then, for each participant, a map was generated indicating the level of evoked activity as a function of each viewed face area. We expected that, unlike what was found in ASD patients by Lio et al. [38], face parts that carry a rich amount of social information, such as the eyes, will yield selective STS responses in WS patients, just as was found.

Second, we performed a single trial analysis with an evoked activity a priori. Using a “facial cue” map as regressor of evoked activity, we investigated how much the multichannel EEG signal was able to decode this predicted pattern of evoked activity at every time point. This analysis has the capacity to indicate at which point in time socially relevant facial features of the regressor induce cortical reactivity.

## Results

### Behavioral results

In the gender discrimination task, WS patients correctly identified 67% of faces while neurotypical subjects and ASD were able to reach 91% and 89% of correct responses, respectively. Although WS patients were less accurate at discriminating gender compared to the other groups (WS vs ASD: *t*(27) = 5.25, *p* < 0.001 and WS vs neurotypical: t(27) = -5.61, *p* < 0.001) they were able to perform above chance-level (*t*(13) = 4.17, *p* < 0.001). Reduced gender discrimination performance in patients with WS may be explained by deficiencies in both general working memory and face processing capacities [39, 40]. The accuracy did not vary as a function of the face part (*F* (24, 975) = 0.7, *p* = 0.85) and face parts did not interact with the group effect (*F* (48, 975) = 0.74, *p* = 0.91).

### EEG results

#### Analysis 1: Spatio-temporal a priori

Group results are shown in Fig. 2. We report the degree of evoked activity in the STS measured as a function of the face region attended by participants with maximum and significant (p < 0.05 Family-Wise Error Rate (FWER) corrected) areas of evoked activity. As reported by Lio et al. [38] in neurotypical subjects, the evoked activity is eye-sensitive. These subjects produced a maximal evoked activity in the upper part of the face (p < 0.05 FWER corrected, indicated by blue tiles over the right small face), over the eyes and eyebrows, and a local minimum in the left and right lower corners, outside the face area. These results replicate the findings of Lio et al. [38] in a young neurotypical population and support the idea that face related signal in the STS is not strongly influenced by the age of participants (see Fig. 2). Although, the maximal evoked activity is above the left eye brow and at the right eye (and at the right from the right eye) which may suggest that a developmental trend could exist.

Patients with ASD showed an atypical pattern with significant activity on the nose region and cheek (p < 0.05, FWER corrected, see Fig. 2, bottom left). These previously reported results are consistent with neurotypical eye tracking behavior in face perception tasks whereby more attention is paid to the eye region than to the mouth areas as well as with previous eye-tracking studies of how ASD patients attend to faces [15, 41]**.**

WS patients showed an activation map with significant activity over the eyes, eyebrows and nose region (p < 0.05, FWER corrected, see Fig. 2, bottom center) and local minimum in the left and right lower corners and outside the face area. The pattern of evoked activity at 260 ms in the STS contrast that of patients with ASD and matches that of neurotypical participants. This result is striking, because we show the existence of a neural process that appears to be preserved and robust in these patients even though the WS population we tested is both younger and has a lower Intelligence Quotient (IQ) than patients with ASD in our sample.

#### Analysis 2: Evoked activity a priori

The maximal evoked activity for neurotypical participants (from [38], from our younger population and from our patients with WS is reminiscent of a T-shape over the face: the eyebrows, eyes, nose and mouth (and these face parts carry rich source of social information), whereas we observe lower activity for other areas of the face that are considered to have little social salience. Consistently, the minimum of the activity was recorded when patients’ eyes were forced to look outside the face. We further built a face cue map spatial regressor generated by applying a sagittal symmetry and subsampling the cortical sensitivity map obtained by the neurotypical population and label this regressor a ‘face cue map’.

Then, for each subject a temporal signal of the Fischer-Snedecor F-statistic denoting the quality of decoding obtained at each time-point between 0 and 1000 ms was generated and compared across groups. The F-statistic allows to evaluate at each time point to what extent the variability of the evoked activity can be explained by the variance modeled by the theoretical 'face cue map’. We report each decoding signal (Fisher-Snedecor F statistic) for each subject. Then, since we want to locate in time the periods where the decoding is maximal, individual F-statistics curves are scaled between zero (min) and one (max) and averaged for the group analysis (Fig. 3).

At the group level, the Fisher-Snedecor F statistic values indicates that the decoding of the signal by the ‘face cue map’ was significant around the 150-350 ms time range after stimulus onset in all three groups (*p* < 0.05, FWER corrected, see Fig. 3 bottom left). The decoding curve in neurotypical yielded two marked peaks, one appearing at 170 ms after stimulus onset, and a second appearing at 260 ms after stimulus onset. This suggests the presence of two independent and/or interacting processes that are dependent on facial cues.

The spatio-temporal dynamics of face processing have been well-studied using intracerebral electrophysiological recordings (e.g., [42]. While late components (after 200 ms) are diffuse in the brain (with peaks in various temporal areas) early components around 160-170 ms have only been identified in the fusiform gyrus, around the FFA. We will therefore assume that the evoked activity modulated by facial features at 170 ms arise from the FFA while the late component that was described by Lio et al. [38] originates from the STS.

Although two response peaks with similar timing were identified in all groups, we observed different response level for these two processes for the two patients’ groups (See Fig. 3A). The ASD population showed a significantly greater decoding peak than the WS population at the earlier timing (170 ms post stimulus onset, ASD > WS – *p* < 0.05 FWER corrected). A reversed pattern can be found at the latter decoding peak: patients with WS showed a significantly greater decoding peak than the ASD population at the later timing (260 ms post-stimulus onset, WS > ASD *p* < 0.05 FWER corrected). Because this method of EEG analysis requires no assumptions regarding cortical source, its use offers a significant advantage over more traditional alternatives, especially when applied to neurodevelopmental disorders.

For each group, we further tested which of the two peaks were maximal at the subject level. The temporal distribution of the timings of maximum peak of decoding can be found in Fig. 3B. These temporal distributions were found to differ between groups (Kruskall-Wallis, *Chi*^*2*^(2, 39) = 11.9, *p* = 0.0026). For neurotypical participants, we found that the distribution of the maximum peak of decoding had a bimodal distribution with a dominance for the second process. This distribution indicates that face decoding goes preferentially through a social decoding in neurotypical participants. ASD patients also showed a bimodal distribution but the early process at 170 ms was significantly prominent. Consistent with behavioral results, this pattern suggests that faces’ related processes within the ventral regions are well-preserved. Finally, WS patients showed a strictly unimodal distribution such that activity was focused around the second process only.

Post-hoc analyses revealed a clear dissociation between both the WS and ASD patients (*p* < 0.01 FWER corrected) and the WS group and neurotypical population (*p* < 0.05 FWER corrected). This result combined with the low performance of WS patients in the gender discrimination task, suggests a preferential use of the dorsal face processing regions during face processing tasks compared to the ventral and is consistent with social cognition biases found in this syndrome.

### Prediction of EEG decoding with behavioural measures

A significant regression equation was found for neurotypical participants *F* (1,13) = 8.56, *p* = 0.013, with an R^2^ of 0.42 to predict decoding timing. Visuospatial reasoning abilities (matrix score, WISC subtest) were the only significant predictor of the maximal onset of decoding (*t* = -2.9, *p* = 0.013). Chronological age and linguistic reasoning abilities (Similarities test, WISC subtest) were not significant predictors. In patients, no significant regression equation was found, most likely due to the lack of variability across participants on the decoding timing in each sample.

As a resume, when examining face processing related neural signals along the occipito-temporal stream, we found two peaks in neurotypical participants: the first at 170 ms, an early signal known to be implicated in low-level face features, the second at 260 ms, a late component implicated in decoding salient face social cues. Remarkably, both components were found distinctly impaired and preserved in WS and ASD. In WS, we could weakly decode the 170 ms signal probably due to their relatively poor ability to process faces’ morphology while the late 260 ms component shown to be eye sensitive was highly significant. The reverse pattern was observed in ASD participants who showed neurotypical like early 170 ms evoked activity but impaired late evoked 260 ms signal.

## Discussion

The present data have theoretical implications for understanding WS, ASD and their characteristic atypical processing of social cues, particularly faces. Indeed, our findings suggest that patients with WS, when viewing the eyes of a face, lack specificity in their early neurophysiological response. Early neurophysiological responses to faces at 170 ms are evoked by facial features [24, 25]. This is thought to allow a consistent description of facial parts to be computed, combined and processed as whole representation using the fusiform face area at 170 ms [23, 26, 27, 43].

We show that early responses in patients with WS are not modulated by the type of facial features on display (i.e., eyes, eyebrow, mouth compared to other face-parts). Such an absence of modulation may suggest that patients with WS make little distinction between facial features and their possible relevance as a means for facial identification and subsequent holistic processing. Indeed, previous studies have already shown abnormal processing at N170 in WS: WS patients show an abnormal increased amplitude at N170 when viewing faces [44, 45].

In the present study, we also show that WS patients produce robust responses to late processing of facial cues at 260 ms in the STS. This observation is consistent with previous fMRI studies reporting greater STS activity in WS patients compared to patients with anxiety disorders while performing a facial perception task [46]. Indeed, the present finding supports the hypothesis that early (170 ms) visual processing of faces in the temporo-occipital area (FFA) and late social processing of faces in the temporal sulcus (i.e. STS) are functionally dissociable as previously suggested using rapid transcranial magnetic stimulation [47].

Our findings support two alternative interpretations: First, the role of the STS, which is typically implicated in late social processing of faces in neurotypical participants [8], may be upregulated in patients with WS, thus leading to their characteristic highly social behavior. A second interpretation may be that early visual activity in temporo-occipital areas is dysfunctional in WS and are thus unable to discriminate specific facial features. This interpretation is supported by previous reports showing that the FFA is both enlarged [48] and structurally altered [49] in these patients.

In our study, the poor decoding of the facial cue regressor is more likely related to their poor visuospatial abilities. In fact, participants who showed poor visuospatial abilities where more likely to exhibit a late peak than those with good performance. This could also explain why WS patients showed a poorer performance on gender discrimination task given their deficits in the perceptual holistic processes of faces. Therefore, it is possible that the first facial process in the ventral part of patients’ brain poorly decode facial features due to poor visuospatial abilities.

Subsequent cascading developmental consequences or compensatory strategies may induce a secondary upregulation of facial processing in other functional structures, namely the STS in this case, thus, leading to a concomitant increase in the social processing of faces. The role of cascading developmental consequences has already been theorized to explain atypical behaviors in other domains associated with Williams’s syndrome (such as mathematical impairments explained by their poor visuospatial abilities, [50]. Such hypothesis is consistent with the atypical visual processing of faces from early infancy in WS [45, 51].

Overall, the notion of cascading pathways between early (170 ms) and late (260 ms) processes remains putative and could be coincidental. However, we argue that their development is closely intertwined. Still, whether the early neurophysiological face processing deficit in WS emerges from the neurodevelopmental consequence of the disorder or as the result of the indiscriminate social interaction exhibited by WS patients remains an unresolved question.

Given that patients with WS in our sample were relatively young (between 8 and 21 yo), it is possible that the atypical response at 170 ms might need more brain maturation and it may change with experience to become similar to that of neurotypical participants in older WS. However, such early adversity could induce long-term effects on visual processing or instead reach neurotypical performance later on, as is the case for face processing behaviour. Indeed, the frequency of pathological gazing at faces in WS individuals decreases after infancy and a more neurotypical focus on the eyes is observed with age [52].

One could see the lower intellectual abilities of patients with WS compared to patients with ASD and age-matched controls as a limitation, however, this ability are most representative from their disorder (i.e., intelligence range from 20 to 106, [53], with a remarkable deficit in visuospatial construction, [54]. Concerning Intelligence Quotient (IQ), most individuals with WS exhibit some degree of intellectual impairment, with the majority of adults scoring in the mild range of intellectual deficiency, especially in the visuospatial domain [55].

Overall, our results raise the possibility of developing functional and behavioural rehabilitative procedures for both WS and ASD available in intellectual deficiency and based on neurofeedback, a procedure in which self-regulation is stimulated by providing online feedback of neural activity to participants [56, 57].

Here, the goal would simply be to manipulate neural activity in the STS or FFA in order to rebalance social processing of face related to this structure, i.e., the identification of facial features. Evoked activity measured by high-density EEG while participants focus on different facial areas (either social, eyes, nose, brows, mouths or not) could be measured and used in real-time to display the decoding level from either the early (FFA, for WS) or late (STS, for ASD) component in real time, through visual, audio or other means, back to participants. The goal of this information would be to help participants self-regulate their cortical excitability related to face processing. Along with cognitive trainings, this procedure might assist patients in reassigning their attention to facial features for the purposes of identity recognition or social cognition [21].

## Conclusions

To conclude, double dissociations using fine spatiotemporal analysis of this network provide essential evidence for understanding what, where and when neurocomputations are performed in our brain. In patients with WS, poor decoding of facial features by low-level visual processing can be associated with over trustiness and hyper familiar approach to strangers that can have pejorative influences on their quality of life and safety. Future works may examine whether and how, in turn subtle changes in the balance of this network, through self-regulated neurofeedback training, can attenuate their social disturbances and related psychiatric symptoms, such as anxiety or depression.

## Materials and methods

### Participants

We recruited 14 neurotypical participants (5 men and 9 women, mean age = 11.6, range = 6–21) and 14 WS patients (5 men and 9 women, mean age = 11.6, range = 6–21) matched for age and gender. The number of participants in each group was selected to provide a balanced designed across groups, therefore we matched the size of groups to that acquired by Lio et al. [38]. Although a larger sample size allows to find a smaller statistically significant difference, the difference found may not be clinically and scientifically meaningful, and furthermore, with respect to patients, we did not wish to submit unnecessary subjects to the procedure [58]. Importantly, as shown by Lio et al., the EEG procedure was meant to be sensitive at the single subject level. We compared the present dataset with the EEG data of 14 ASD patients (14 men, mean age = 20, range = 18–21) recruited in a previous study by Lio et al, [38], Exp. 3). All participants had normal or corrected to normal vision and all neurotypical participants had no history of psychiatric or neurological disease.

Participants were recruited through national advertisements from WS associations and from the Reference for Rare Diseases Lyon Center (Vinatier hospital, C.D.). Each patient received a diagnosis of WS following genetic assessment (deletion at 7q11.23) by the psychiatrist (CD) involved in the study. Patients with WS and neurotypical subjects both participated in a short neuropsychological evaluation to assess visuospatial reasoning, logical thinking and verbal skills (matrix and similarities subtest, WISC-V [59] and visuospatial and auditory attention (Arrows and auditory attention subtest, NEPSY, [60]. Unsurprisingly, patients with WS showed lower performances compared to controls in all of these tests (see Table 1). These subtests were selected because they strongly correlate with the general IQ (WISC-V, [59], and thus, allow us to determine the intellectual deficits of each patients with WS. As expected, scores from the control group were in the normal range. Neuropsychological data of ASD patients obtained from Lio et al.’s study involved IQ evaluation (WAIS III or WAIS IV). Patients with ASD showed normal intellectual abilities (Mean IQ = 97.20, SD = 27) with the exception of two patients showing lower scores on these tests (IQ < 75) and two patients showing high scores on these tests (IQ > 125) responsible for the high standard deviation of the group (See Table 2).

**Table 1:** General cognitive abilities assessed in neurotypical participants and patients with WS. Mean represent scaled scores for Wechsler substests (norm: mean = 10; SD = 3)

General cognitive abilities tests	Neurotypical (N = 14)	Patients with WS (N = 14)	Two Samples t-test *P*-value
	Mean (SD)	Mean (SD)	
Matrices	11.9 (2.8)	3.8 (2.9)	< 0.001
Similarities	15.3 (2.7)	4.5 (3.4)	< 0.001
Arrows	12 (2.5)	2.1 (2.1)	< 0.001
Auditory attention	10.5 (4.0)	6.5 (5.2)	0.035

**Table 2:** General cognitive abilities assessed in patients with ASD. Mean represent scaled composite score for Wechsler index (norm: mean = 100; SD = 15)

General cognitive abilities tests	Patients with ASD (N = 14)	Significant difference to the norm
	Mean (SD)	
Total IQ	97.2 (27)	NS
Verbal IQ	98.4 (21)	NS
Non Verbal IQ	104.6 (28)	NS

The ability to recognize facial cues such as those related to emotional states is known to improve with chronological age. Significant changes in this ability have been found to occur in the first two years of life (for a review, [61] and to have a strong influence on social experience [62]. Chronological age, however, does not correlate with social approach ratings in Williams syndrome [63]. Changes in the neural network dedicated to face processing are nevertheless related to chronological age (e.g., for the FFA, [64]; for the STS, [65]).

Here, we used chronological age as a control basis for the amount of face-to-face experience participants accumulated and as an index of expected maturation processes in the face processing network. Experimental protocols based on intellectually age-matched designs are indeed known to be limited when dealing with population with intellectual disability, especially those with an heterogeneous profiles such as WS [66]. Although WS patients are matched on global intellectual abilities, they are known to face more challenge in the visuospatial domain than in the verbal domain compared to other neurodevelopmental disorders. Therefore, we decided to control for the effect of chronological age by comparing patients with WS with an additional group of age-matched neurotypical participants and to statistically assess the existence of a mental age bias in our neurotypical population. To do so, we asked whether the intellectual abilities and chronological age of neurotypical participants (assessed with the matrix and similarities subtests) contribute to the observed effects in the EEG activity (see the section “Prediction of EEG decoding with behavioural measures”).

The study was approved by the French Sud-Ouest Lyon Bérard ethical committee (project N ° 2018-A02037-48). All methods were carried out in accordance with relevant guidelines and regulations. Prior inclusion, all participants and/or their legal representative provided written informed consent to participate in the study.

### Procedure

**Fig. 1:**
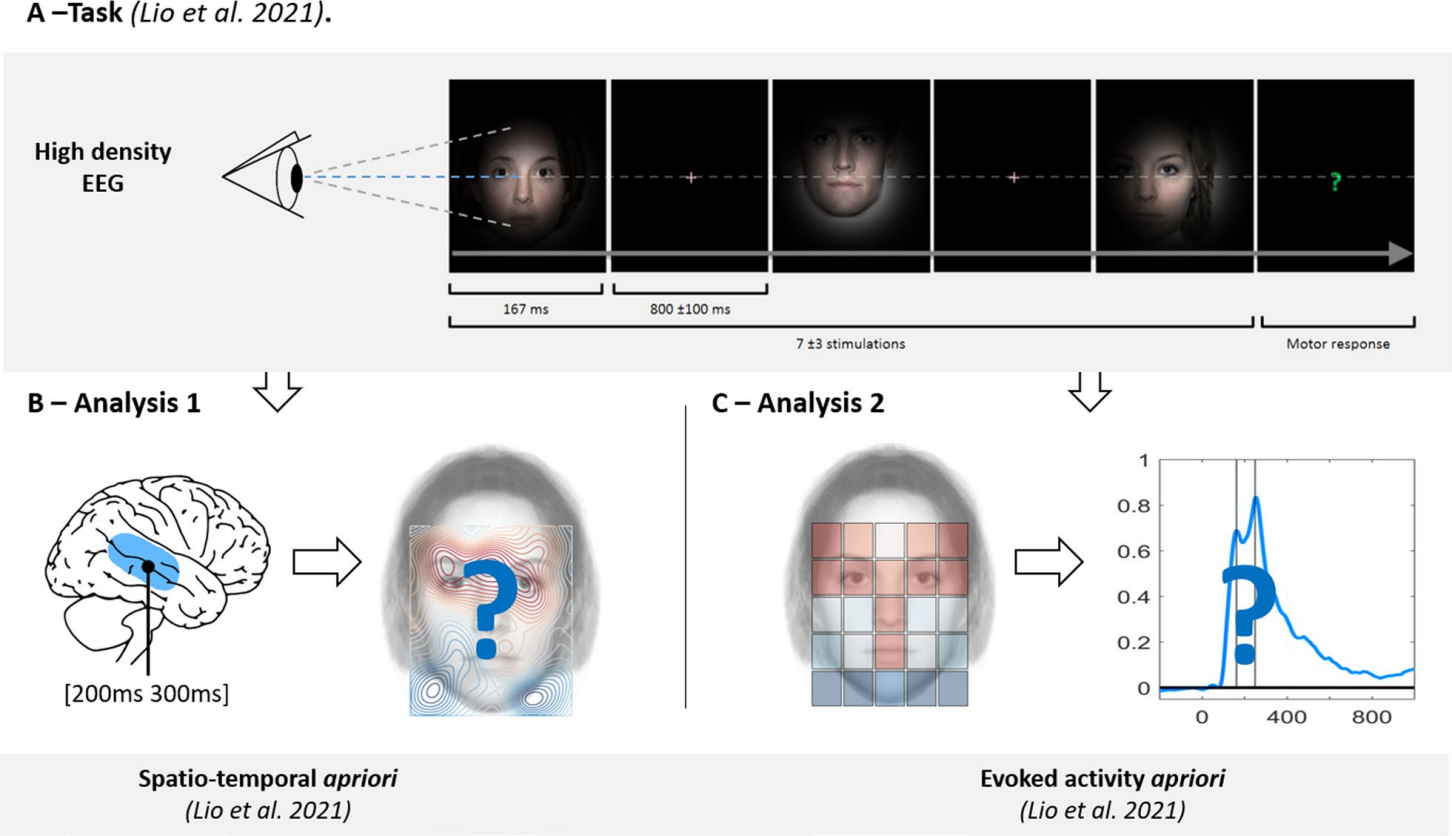
Methodological procedure summary

Participants were instructed to focus on a fixation cross (with a duration jittered 800 ± 100 ms), which was followed by a face stimulus masked by a Gaussian apodization window (a Full Width at Half Maximum (FWHM) = 10°) centered on the fixation cross that appeared for 167 ms (Fig. 1A). Using this procedure, we were able to control which face region subjects were focusing on and the luminance distribution projected on to the retina. This procedure which reduces eye movements is similar to the “bubbles approach” method for studying the unit of face information processing [67]. To maintain subjects’ attention, every 7 (± 3) trial would take the form of a question mark which was presented on the screen instead of a face stimulus. Upon presentation of these trials, participants were required to recall the gender of the last face displayed and to respond using a button press with either their index or middle finger. The focus was on accuracy rather than speed as behavioural response was not the main goal of the study. Experimental testing was made up of three sessions each consisting of 500 trials. This was preceded by a training session with 100 additional trials to ensure that the gender discrimination task was performed above chance-level and that all participants understood the instructions. We set out to control the area of the face focused on by the participant in order to link it to cortical reactivity occurring at 200-300 ms. Furthermore, we presented a limited number of trials to participants to ensure that the duration of task performance did not exceed an hour. This ensured that the task would be more amenable to the attentional abilities of young participants and patients with WS. Following pilot testing with adult participants with WS, we selected a total of 1500 face presentation trials (plus the 100 training trials).

### Stimuli

Face stimuli were identical to those used by Lio et al [38], Exp. 3) and delivered using Matlab and the Matlab Psychtoolbox. Each stimulus consisted of a neutral natural face controlled in proportion, position and luminance distribution. The faces are displayed on the screen at different locations on a grid, drawn from a uniform distribution. Then, each stimulus is multiplied by a Gaussian apodization window centered on the fixation cross. With this procedure, each trial consists of projecting different parts of the face onto the subject's fovea while controlling the luminance distribution of each stimulus. The width of the aperture window was chosen so that it was large enough (Full Widths at Half Maximum = 10° of visual angle) to make the recognition of gender or the identity of each stimulus easy across all trials (Fig. 1A. Considering, the coordinate [0°; 0°] located between the two pupils of face pictures, each observed region is drawn from a uniform distribution on a [− 8°; + 8°] visual angle width, [− 12°; + 8°] height rectangle encompassing the whole face area. Thus, with this method, each region of the face is observed multiple times by the subject. In this study, in order to optimize the statistical power of the analysis given the reduced number of trials (1500, the region of focus for each stimulus presentation was no longer randomly selected from all of the pixels in the picture (20° height × 16° width, as in Lio [38], exp 2). Instead, regions were centered on one out of 25 rectangles defined as ROIs. These ROIs were generated by dividing the overall area of the studied face stimuli into a grid of 5 × 5, each ROI took the form of a rectangle (size, height = 4° x width = 3.2°). Given that we presented and recorded 1500 trials, the sampling density of face stimuli was 1500/25 = 60 trials/ROI/participant.

### EEG recording and preprocessing

We used the Brain Product™ actiCHamp system to record the electroencephalographic signal from 128 active electrodes (actiCAP 128Ch Standard-2) mounted in an elastic cap at 10–10 and 10–5 system standard locations [68]. All electrode impedances were kept below 50 kOhms. Subjects were seated in a darkened, shielded room with their head position controlled by an ophthalmic chin-rest device so that eye-level was aligned with the fixation cross. EEG data were recorded at a sampling rate of 5000 Hz with an online reference at the Fz electrode. Offline, data were band pass filtered using zero-phase Chebychev type II filters (Low pass—cutting frequency: 45 Hz, transition band width: 2 Hz, attenuation: 80 dB; order: 35, sections: 18 | High pass – cutting frequency: 0.3 Hz, transition band width: 0.2 Hz, attenuation: 80 dB; order: 9, sections: 5) and re-referenced to a common average. Next, data were epoched from 200 ms before to 400 ms after the stimulus onset.

Traditional EEG analysis considers the time course of individual channels. However, with modern high spatial density EEG (128 electrodes), we used multivariate signal processing algorithms as it can linearly combine channels to generate and aggregate representation of the data. This linear projection combines the information from the multiple sensors (128) into a single channel whose time course can be analyzed with conventional methods (temporal filtering, trial averaging), thus improving the spatial resolution and signal to noise ratio compared to traditional analysis [69, 70].

### Analysis 1: Spatio-temporal a priori

First, a spatial filter allowed the extraction of single-trial activity evoked between 200-300 ms in the STS (Fig. 1B). This filter relies on the scalp topography of the source as established by Lio et al. [38] (See section below for more details). Then, for each participant, a map was estimated of the level of evoked activity as a function of the focused face area. Finally, for each group, an average was generated that estimated which face area evoked the most activity.

### Single trial spatial filtering

Using a Group Blind Source Separation (gBSS) analysis and cluster-permutation test Lio et al. [38], revealed a significant source in neurotypical participants consisting in a large evoked activity with a maximum at 240 ms after the stimulus onset, and a source localization showing a maximum of activity around the lateral fissure (MNI coordinates = X: + 65 -65 Y: − 20 Z: 10) and a local maximum around the inferior temporal sulcus (MNI coordinates = X: + 60 -60 Y: -40 Z: − 20).

Based on the scalp topography of this source, which occurs between 200 and 300 ms, we use spatial filtering to extract single-trial evoked activity in all participants. More specifically, to measure the evoked activity of the component identified in the Lio et al. [38] study, we calculated a spatial filter for each trial using minimum variance beamforming [71, 72] in combination with the spatial information estimated at the group level in the original study using gBSS (group Blind Source Separation) [38], see also [73] for a detailed description of the method).

**Fig. 2:**
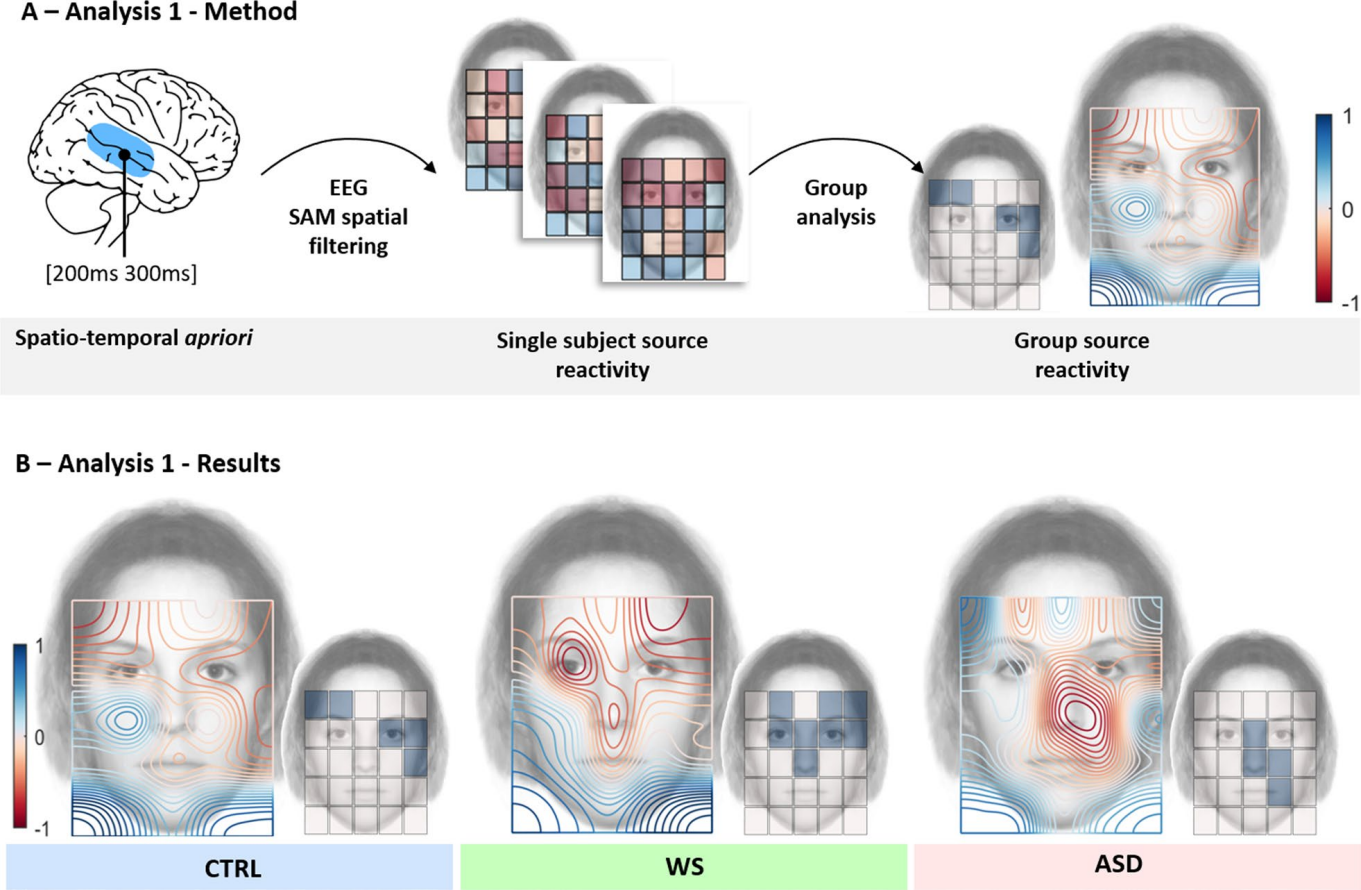
Method and results of the single trial evoked activity analysis. **A** Signal processing pipeline of this analysis 1. First, a spatial-filter was built to extract single-trial activity evoked between 200 and 300 ms in the superior temporal region at the single subject and group-level. **B** Results of evoked activity mapped over a face area for the neurotypical participants (CTRL, in blue, on the left), patients with Williams-Beuren syndrome (WS, in green, in the middle); and patients with autistic spectrum disorders (ASD, in red, on the right). For each group, the large face on the left (with a Red-Blue color scheme) shows a contour plot of interpolated results of the degree of evoked activity in the STS measured as a function of the face region attended to by participants. The small face on the right (with blue tiles) shows tiles which represent maximum and significant (p < 0.05 FWER corrected) areas of evoked activity. Activity in young neurotypicals confirms that activity was eye-sensitive (significant on the left eye), as found previously in adults (Lio et al. [38] Exp. 2). This was maximal in the upper part of the face, over the eyes and eyebrows, and decreased to reach a local minimum in the left and right lower corners, outside the face area. WS patients showed a similar activation map with significant activity over the eyes, eyebrows and nose region. ASD showed an atypical pattern with significant activity on the nose region and cheek

For each participant, a map was generated indicating the relative level of evoked activity as a function of each of the 25 viewed face areas. To do so, we first average the evoked activity between 200 and 300 ms at each location, then we applied a Z-transform of the 25 obtained values in order to highlight the ‘most positive’ and the ‘most negative’ areas (See Fig. 2A). This analysis allowed us to visualize for each participant how cortical sensitivity in the STS occurring at 200-300 ms is affected by different face regions.

### Group-level

Finally, we studied in each group of 14 subjects, which face region evoked a significantly the more pronounced activity by performing group statistical analyses on the 25 locations (25 non-parametric, N = 14, one tailed, sign tests, p < 0.05, FWER controlled using the maxT/minP multiple testing procedure ([74], Fig. 2). This process led to a statistical non-parametric mapping of the evoked activity in the superior temporal region (Fig. 2B) for each group. For visualization purposes only, we processed a smoothed representation of the results obtained with an original resolution of 5 × 5 using bicubic interpolation of single-subject results and averaging interpolated maps at the group level (Fig. 2B).

### Analysis 2: Evoked activity a priori

First, we generated a “facial cue” map regressor from neurotypical group data provided by Lio et al. [38]. Then, for each participant, we applied a multiple linear regression model to assess how much the multichannel EEG activity was able to decode the “facial cue map” over time (Fig. 1C).

### Facial cue map regressor

**Fig. 3:**
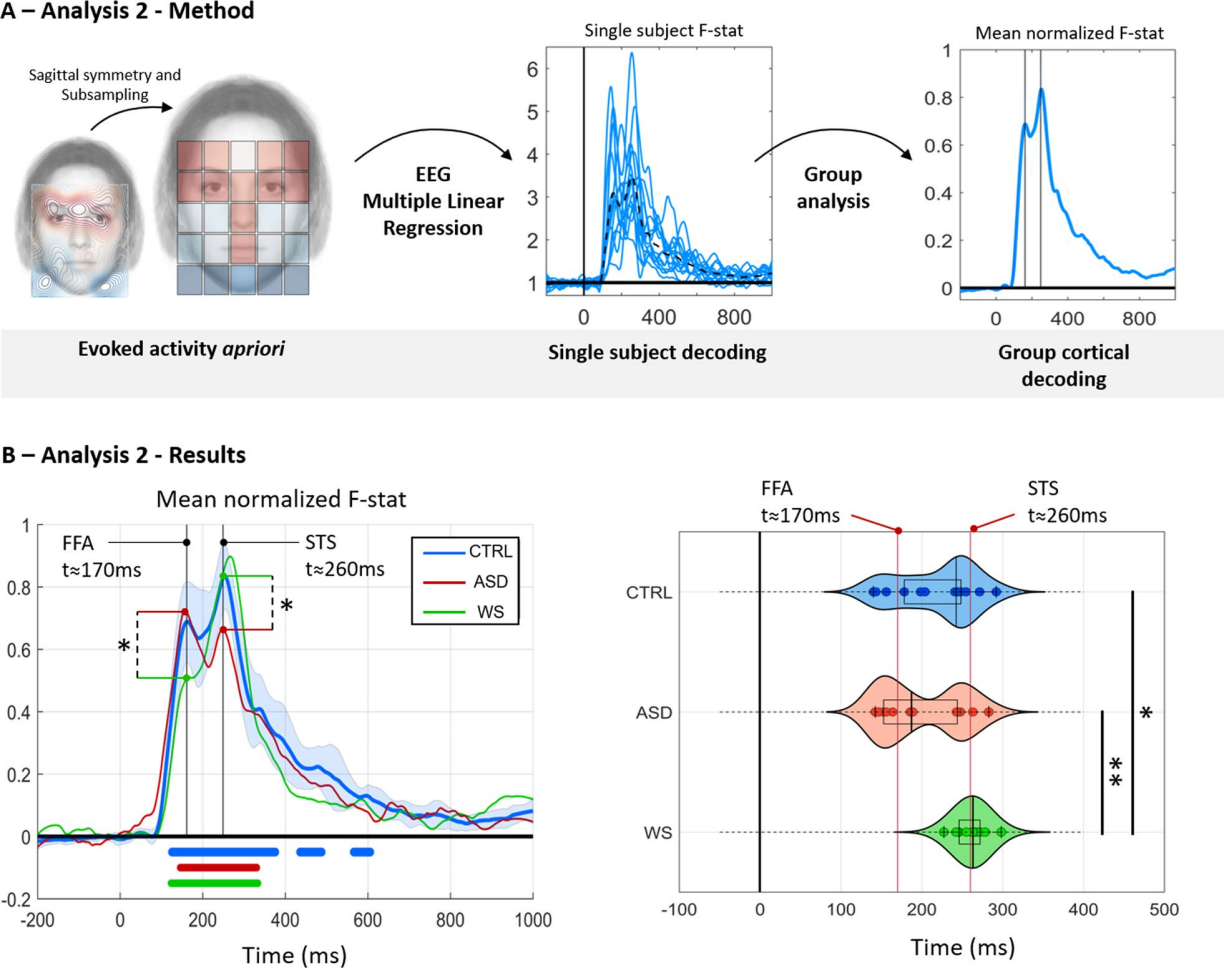
Prediction of evoked activity with face cue map spatial regressor. **A** Signal processing pipeline for the second analysis. The ‘socially relevant face cue map’ regressor was generated from the neurotypical evoked activity (applying a sagittal symmetry and subsampling) (left part). Then, a multiple linear regression is applied and the Fischer-Snedecor F statistic denoting the quality of decoding obtained at each time point between 0 and 1000 ms is generated for each subject (see dotted lines, on the middle graph). Finally, each decoding signal (Fisher-Snedecor F statistic) scaled between zero (min) and one (max) for each subject was averaged for the group analysis (right graph). **B** Results from decoding the facial-cue map for the neurotypical participants (CTRL, in blue), patients with Williams-Beuren syndrome (WS, in green) and patients with autistic spectrum disorders (ASD, in red). On the left graph, mean normalized F score for the decoding of each group as a function of time. Significant Fisher-Snedecor F values at the group-level are underlined in blue, green and red for neurotypical, patients with WS and Patients with ASD respectively. At the group level, the neurotypical decoding curve (blue) presents two marked peaks, one at 170 ms post stimulus onset, the second at 260 ms post stimulus onset. The ASD population (red curve) produced a significantly higher decoding peak than the WS population (green curve) at the earlier timing (170 ms–p < 0.05 FWER corrected). A reversed pattern was found at the latter decoding peak (260 ms post-stimulus onset, WS > ASD p < 0.05 FWER corrected). On the right graph, the distribution of the timing of the maximum peak for each individual in each group is plotted over time. The distribution in the neurotypical population (blue) and in patients with ASD (red) is bimodal with a dominance at 260 ms for neurotypical participants and at 170 ms for patients with ASD. The distribution in WS patients is strictly unimodal

We generated the “facial cue” map regressor by applying a sagittal symmetry and subsampling the cortical sensitivity map generated by neurotypical participants as in Lio et al.’s study [38] (See Fig. 3A left). The obtained regressor implies that evoked activity is maximal in the eyes and eyebrows region and gradually decrease over the nose and mouth and other face regions to reach a minimum outside the face.

### Multiple linear regression of EEG activity

With this analysis, we avoided making any spatial modelling assumptions with regard to sources or anatomy and relied entirely on the statistics of the observed data and its covariation with observable stimuli [70]. Here, we denote x(*t*) as the vector of multidimensional EEG data (from all the 128 channels from the recording) at time *t*. A linear projection combines the information from the multiple sensors (128) into a single channel y(t), whose time course can be analysed with conventional methods [70]. A vector w(t) is selected or calculated based on constraints or desired attributes of the time series y(t). Her, we aimed to find a weighted matrix w(t), at each time points, that could discriminate at the single-trial the level of evoked activity expected for the part of the face presented.

A multiple linear regression model was calculated at every time point, to predict the facial cue map regressor Y based on the multichannel EEG activity x (t): Y = w(t) × x (t) + n(t). Significant regression equation is reported using the Fisher-Snedecor (F) statistic, at every time point (between 0 and 1000 ms), which represents how the quality of the decoding signal or how the EEG evoked activity at t codes for the facial cue map regressor. The Fisher-Snedecor (F-stat) is scaled between zero (min) and one (max) for each subject and averaged for the group analysis.

For each individual, we collected the maximum timing peak of the Fisher-Snedecor F statistic. We tested if the temporal distribution of the maximum peak differed across groups (Neurotypical, ASD, WS) using a Kruskall-Wallis test. To account for potential behavioural confounding, we performed a multiple linear regression to determine whether the variability of the onset of peak decoding was dependent on behavioural variables. Specifically, we performed a multiple linear regression (stepwise) to predict when peak decoding occurs as a function of task accuracy, visuospatial reasoning ability (matrix), linguistic reasoning ability (similarities) and age. In ASD patients, the linear regression was performed using the verbal and the Performance IQ as regressor, instead of the visuospatial and language reasoning abilities scores.

## Data Availability

The conditions of our ethics approval do not permit public archiving of patients’ data. Public access to data can be obtained after permission of the French ethical committee (CPP Sud-Est IV) who granted ethical approval to this project (N°CPP:16/018, N° ID RCB: 2014-A01894-43, Promoted by CNRS).
